# The COVID-19, tuberculosis and HIV/AIDS: Ménage à Trois

**DOI:** 10.3389/fimmu.2023.1104828

**Published:** 2023-01-27

**Authors:** Aniefiok John Udoakang, Alexandra Lindsey Djomkam Zune, Kesego Tapela, Nora Nghochuzie Nganyewo, Frances Ngozi Olisaka, Claudia Adzo Anyigba, Sylvia Tawiah-Eshun, Irene Amoakoh Owusu, Lily Paemka, Gordon A. Awandare, Peter Kojo Quashie

**Affiliations:** ^1^ West African Centre for Cell Biology of Infectious Pathogens (WACCBIP), College of Basic and Applied Sciences, University of Ghana, Accra, Ghana; ^2^ Department of Biosciences and Biotechnology, University of Medical Sciences, Ondo, Nigeria; ^3^ Department of Biochemistry, Cell and Molecular Biology, College of Basic and Applied Sciences, University of Ghana, Accra, Ghana; ^4^ Medical Research Council Unit, The Gambia at the London School of Hygiene and Tropical Medicine, Banjul, Gambia; ^5^ Environmental and Public Health Microbiology, Department of Biological Science, Benson Idahosa University, Benin City, Edo State, Nigeria; ^6^ Department of Ophthalmology, Korle-bu Teaching Hospital, Accra, Ghana; ^7^ Virology Department, Noguchi Memorial Institute for Medical Research, University of Ghana, Accra, Ghana

**Keywords:** COVID-19, HIV/AIDS, tuberculosis, co-infection, pandemic, susceptibility, immunosuppression

## Abstract

In December 2019, a novel pneumonic condition, Coronavirus disease 2019 (COVID- 19) caused by severe acute respiratory syndrome coronavirus 2 (SARS-CoV-2), broke out in China and spread globally. The presentation of COVID-19 is more severe in persons with underlying medical conditions such as Tuberculosis (TB), Human Immunodeficiency Virus/Acquired Immunodeficiency Syndrome (HIV/AIDS) and other pneumonic conditions. All three diseases are of global concern and can significantly affect the lungs with characteristic cytokine storm, immunosuppression, and respiratory failure. Co-infections of SARS-CoV-2 with HIV and *Mycobacterium tuberculosis* (*Mtb*) have been reported, which may influence their pathogenesis and disease progression. Pulmonary TB and HIV/AIDS patients could be more susceptible to SARS-CoV-2 infection leading to lethal synergy and disease severity. Therefore, the biological and epidemiological interactions of COVID-19, HIV/AIDS, and TB need to be understood holistically. While data is needed to predict the impact of the COVID-19 pandemic on these existing diseases, it is necessary to review the implications of the evolving COVID-19 management on HIV/AIDS and TB control, including therapy and funding. Also, the impact of long COVID on patients, who may have this co-infection. Thus, this review highlights the implications of COVID-19, HIV/AIDS, and TB co-infection compares disease mechanisms, addresses growing concerns, and suggests a direction for improved diagnosis and general management.

## 1 Introduction

The novel coronavirus disease 2019 (COVID-19) was declared a pandemic by the World Health Organization (WHO) on March 11, 2020 (1). It is the third major viral pandemic after the Human immunodeficiency virus/acquired immunodeficiency syndrome (HIV/AIDS) and the Spanish flu (2, 3). The COVID-19 is caused by the novel Severe Acute Respiratory Syndrome Coronavirus 2 (SARS-CoV-2), which has infected more than 620 million people globally, including approximately nine million Africans (4). Transmission of SARS-CoV-2 occurs primarily through infected saliva and respiratory secretions or droplets (5). Symptoms of COVID-19 include fever, cough, general malaise, and dyspnea, while hypertension and diabetes as the most common underlying comorbidities (6–9). Also, some COVID-19 patients experience severe disease, which is characterized by acute respiratory distress syndrome (ARDS) and lung injury due to a damaged alveolar lumen (9–11). Others have cognitive and neurological problems due to post-COVID-19 conditions termed long COVID-19 conditions (12).

Secondary or co-infections, with bacteria (*Mycobacterium tuberculosis* (*Mtb*)), fungi, and viruses (influenza, coronavirus, HIV, metapneumovirus), have been observed in some COVID-19 patients (13–15) with varying incidences and about 50% secondary infections in non-survivors (15–22). Co-infections with SARS-CoV-2 contribute significantly to disease morbidity and mortality (22) and people living with HIV (PLWH) are four times more likely to experience post-COVID-19 conditions (23). Post-COVID-19 conditions known as the ‘‘Long-term effects of Coronavirus’ are persistent symptoms and consequences that developed after one month from COVID-19 diagnosis (12, 24). People of low socioeconomic status, those having pre-existing conditions and unvaccinated individuals have a higher risk of developing long COVID-19 (25, 26). This is of grave medical and socio-economic concern, especially where tuberculosis (TB) and HIV/AIDS burdens are high such as in Africa. Understanding host-pathogen biology and geographical variations of co-infection with TB and HIV are of utmost importance. Therefore, this review aims to highlight the effect of COVID-19, HIV/AIDS, and TB co-infections and address growing concerns. The information will contribute to COVID-19, HIV/AIDS and TB management, especially in Africa.

## 2 The burden of HIV/AIDS and TB co-infection in Africa

**Figure 1:**
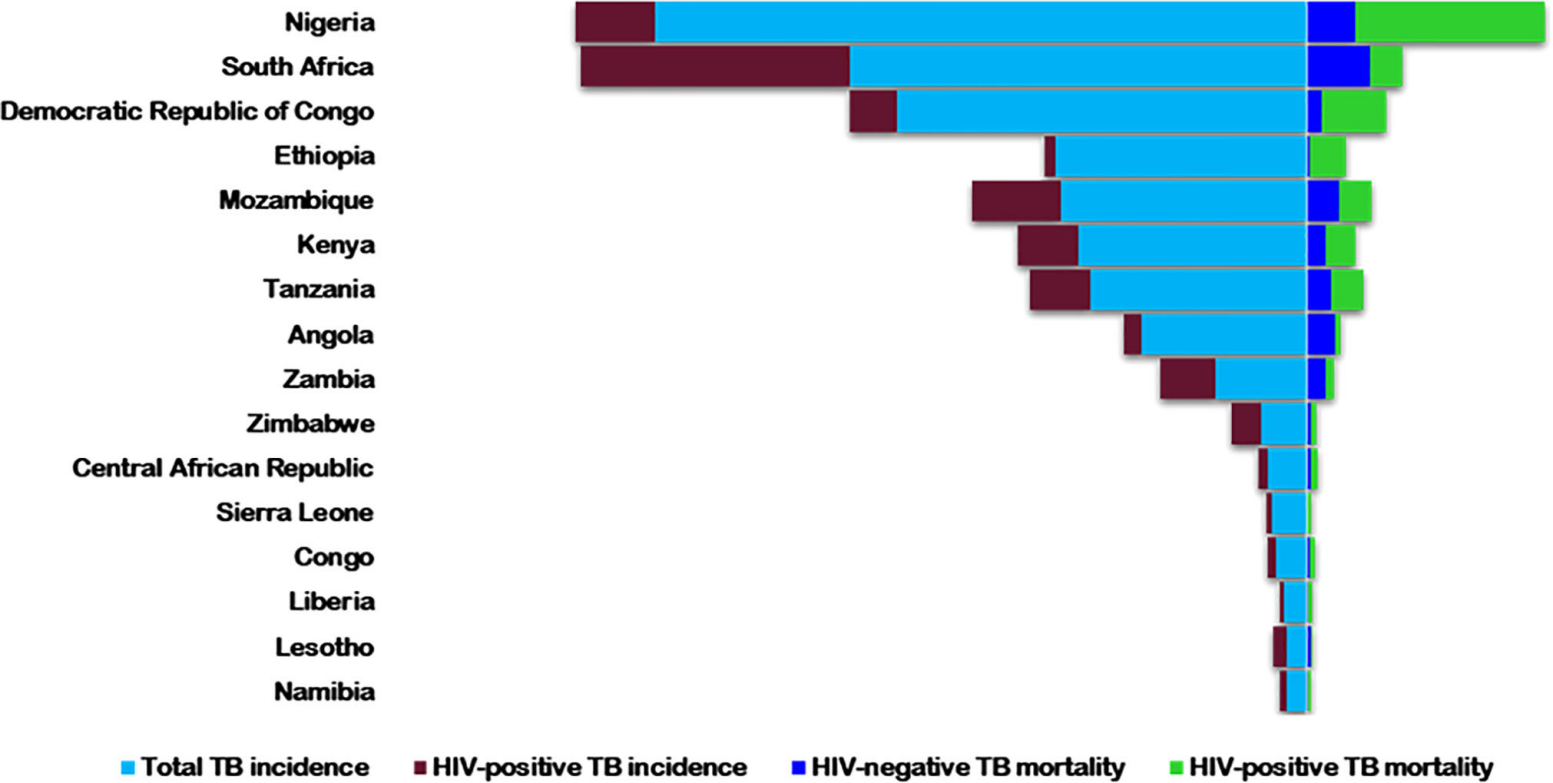
The 2018 estimated rates of HIV+/-TB incidence and mortality in the highest TB/HIV buden countries in Africa. Rates are per 100,000 populations [World Health Organization, Globa; Tuberculosis Report 2019(38)].

HIV is a comorbid infection in COVID-19 patients (27). It has infected about 74.9 million people globally, with more than 32 million deaths from AIDS-related illnesses (28). In 2021, about 38.4 million people were living with HIV (PLWH), with more than half in Africa (28). Tuberculosis, despite being preventable and treatable, is still one of the major causes of death, especially in PLWH - accounting for about one-third of global deaths (29). Globally, HIV/AIDS and TB are collectively the two most deadly infectious diseases and are tragically interconnected (30). As HIV suppresses the immune system, it predisposes patients to *Mtb* infection, hastens progression to active disease, and increases latent TB reactivation by 20-folds (31, 32). Alone, TB was the leading cause of death from a single infectious pathogen, before the COVID-19 pandemic (33), and the ninth leading cause of mortality globally (34). Africa has a high TB infection burden - 25% of the global new cases and deaths - but the lowest reduction rate worldwide (34). Twenty-two of the 30 high-burden HIV/TB countries are in Africa (35) (**Figure 1**).

The interaction between HIV/AIDS and TB presents a “toxic negative synergistic effect” mostly impacting people within impoverished regions and causing high mortality (36). Therefore, triple infection with SARS-CoV-2, HIV, and *Mtb* is concerning as evidence and experience from other viruses and chronic illnesses show that HIV/*Mtb* co-infected patients have a greater risk of severity with secondary infections (37).

### 2.1 COVID-19, TB and HIV/AIDS: Cytokine storm syndromes and immunosuppression

Cytokine storm is a dysfunctional release of cytokines in response to infections or external stimuli, resulting in acute/severe inflammation and multiple organ damage (38). This complex mechanism involves loss of regulatory control of pro- and anti-inflammatory cytokine production, at local and systemic levels (39). Elevated levels of pro-inflammatory cytokines, including interleukin-1β (IL-1β), IL-8, IL-6, CXC-chemokine ligand 10 (CXCL10), and CC-chemokine ligand 2 (CCL2) have been observed in severe COVID-19 patients (40) and the profuse inflammatory responses during SARS-CoV-2 infection promote additional unrestrained pulmonary inflammation, likely a prime cause of case mortality (41). Rapid viral replication and cellular damage, virus-induced Angiotensin-Converting Enzyme 2 (ACE-2) downregulation and shedding, and antibody-dependent enhancement (ADE) are accountable for the hostile SARS-CoV-2-mediated inflammation (42). The debut rapid viral replication can lead to massive epithelial and endothelial cell death and vascular leakage, triggering excessive pro-inflammatory cytokines production (43). Additionally, increase Angiotensin II levels in SARS-CoV-2 infection secondary to ACE-2 down-regulation, triggers NF-κB and IL-6-STAT3 inflammatory pathways, particularly in non-immune cells, including endothelial and epithelial cells (44). This pathway forms the IL-6 amplifier positive feedback cycle, resulting in excessive activation and cytokine storm (44) leading to vascular permeability and ARDS.

The intricate interaction between *Mtb* and the immune system is associated with the release of cytokine array, including IL-6, IL-10, IL-12, IL-17, IL-18, IL-22, IL-23, IL-27, TNF-α, IFN-γ, IFN-β, IL-1β, and TGF-β, secreted by different cell types (45, 46). Excessive pro-inflammatory cytokines release, leading to cytokine storm, has been associated with lung damage and TB severity. However, *Mtb*-induced cytokine is a “*circular model*” that balances pro- and anti-inflammatory cytokines (46). Studies have suggested that pulmonary TB and COVID-19 co-infection can likely cause prolonged respiratory symptoms, fever and unresolved radiological abnormalities (47). Latent TB patients exposed to SARS-CoV-2 produce increased cytokines and chemokines levels associated with both resistance and susceptibility to active TB disease (48) suggesting induced activation of latent TB by COVID-19 when the balance between active TB resistance and susceptibility cytokines tips towards susceptibility.

HIV infection concords with immune activation, exhibited by the increase of several plasma cytokines (49–51), specifically seen at early infection. Acute HIV infection is distinguished by the onset of cytokine storm preceding peak viremia (50–52). At the viremia peak, the cytokine storm partially resolves with persistent elevation of some cytokines such as TNF, IFNɣ, IL-22, and CXCL10 above pre-infection physiologic levels and into the chronic phase in untreated infections (52). Although anti-retroviral therapy (ART) greatly lowers the plasma levels of inflammatory cytokines like CXCL10, residual immune activation prevails despite early ART initiation (53–55). While T cell activation during early infection is known to influence subsequent disease progression (56), plasma cytokine production during acute infection defines the disease prognosis (57).

Cytokine storm occurs early in HIV infection contributing to disease progression; however, it occurs late in SARS-CoV-2 infection constituting an aspect of disease progression. Therefore, the anti-inflammatory effects of immunosuppression could be protective to alleviate poor outcomes in cytokine storm-related COVID-19 patients (58). Hence, it will be interesting to know if HIV has a protective advantage against severe forms of COVID-19. Several studies claim an overall asymptomatic or mild COVID-19 course in immunocompromised patients, including children undergoing anticancer therapy (59), immunosuppressive chronic drugs users (60), transplant recipients (61), and poorly controlled HIV patients (62). In these studies, a handful of severely affected individuals recovered, and low fatality rates were recorded.

Given the high cytokine levels induced by SARS-CoV-2, inflammation-related lung damage treatment is critical. However, interventions to reduce inflammation may negatively affect viral clearance, which could prevail as sub-clinical infection in the host (63). Similarly, immune-suppressive conditions that reduce inflammatory response have been reported to enhance prolonged virus shedding even after symptoms have resolved (64). This effect of immune suppression on delayed viral clearance can allow for the accumulation of mutations driving the emergence of new SARS-CoV-2 variants. Therefore, the need for clinicians to be careful when assessing COVID-19 in immune-compromised persons, including HIV patients since they could likely experience prolonged shedding of infectious SARS-CoV-2. More clinical studies are needed to find a balance between immune activation and inflammation inhibition.

Although SARS-CoV-2, *Mtb*, and HIV are individually involved in lung pathologies, the kinetics of cytokine storm in each infection is different. It is imperative to understand these differences, especially in regions with the likelihood of co-infections. This will provide specific guidelines for the management of patients in communities where these infections co-exist.

### 2.2 Ménage à Trios: COVID, TB, and HIV/AIDS

Apart from the global economic burden, the COVID-19 pandemic has greatly impacted countries burdened with HIV/TB co-infection, reversing gains made within the past decade (33). Few cases of TB/HIV/COVID-19 triple infection have been reported with the need for hospitalization in several instances (65) while a case of triple infection with extra-pulmonary TB, HIV and COVID-19 was successfully treated without complications (66). SARS-CoV-2 is known to primarily enter cells by binding to the ACE-2 receptor using its envelope spike (S) glycoprotein (67). However, the CD147 (basigin) receptor is an alternative route of SARS-CoV-2 infection *via* Cyclophilin A (CyPA)/CD147 interaction (68). This interaction plays a vital role in the ability of SARS-CoV- 2 to enter the host cells *via* the binding of SARS-CoV-2 S-protein (CD147-S protein) to the CD147 surface molecule (69).

The CD147 similarly enhances HIV infection (69) while CyPA expressly incorporates into HIV-1 virions, significantly enhancing an early cellular infection. In HIV and SARS-CoV-2 infection, the viral protein binds to CD147 together with CyPA. The question here is, what does it mean for the host cell if they are infected with the two different types of viruses? Therefore, the need to investigate co-infections at the molecular level for more insight and understanding of the ‘ménage à trios.

Interestingly, studies have revealed that HIV infection in hospitalized COVID -19 patients is not an imperative comorbid factor (70) as admission rates, morbidity, and mortality in HIV/COVID-19 co-infections are similar to rates seen in the general population (71). Similarly, SARS-CoV infection was reported to cause momentary repression of cellular immunity, leading to further susceptibility and exacerbating reactivation of new *Mtb* infection (72). Therefore, the risk of severe COVID-19 or death in PLWH is lower than perceived and the prognosis better for people on ART (73, 74), while SARS-CoV-2-infected HIV patients without ART are more likely to be hospitalized and die (75). In a study assessing COVID-19 risk in PLWH, administration of nucleotide reverse transcriptase inhibitors, tenofovir disoproxil fumarate (TDF)/emtricitabine (FTC), tenofovir alafenamide (TAF)/FTC, lowered COVID-19 risk and related hospitalization in West and Central Africa, while HIV-positive men older than 70 years had a greater risk of contracting COVID-19 (76). This suggests that TDF/FTC prevents COVID-19 in PLWH by inhibiting RNA-dependent RNA polymerase (RNAdRNAp) (76). Infection with SARS-CoV-2 enhances T-cell exhaustion in PLWH, especially in those without proper ART, leading to severe outcomes compared to patients on ART (77). However, cases of non-fatality (78) imply that HIV infection regardless of ART may not be a risk factor for COVID-19 severity/fatality. Since HIV replication gradually destroys the naive and memory CD4+ T-lymphocytes, incapacitating the immune system (79), low CD4 counts due to COVID-19/HIV co-infections could result in reduced immune response contributing to less COVID-19 complications (73).

The majority of COVID-19 cases are asymptomatic; hence, a primary HIV infection could activate, that is, increase the severity of most asymptomatic COVID-19 cases. Also, the two HIV types, HIV-1 and HIV-2, with differential aggressiveness, could impact the severity or symptomatology of the disease. Nonetheless, the link between HIV and SARS-CoV-2 remains unclear (80), despite reports of significant COVID-19 deaths in immunosuppressed patients (81). More data tying the latter effects of this triplet are needed to understand the underlying impact of co-infection, especially with increased reports and complications of post-COVID-19 conditions.

Generally, the long-term pulmonary sequelae in post-TB patients are well described but not fully characterized in post-COVID-19 patients. Furthermore, the experience of SARS-CoV-2 infection in TB patients remains limited and poor treatment outcomes are predicted in people with both, especially if TB treatment is interrupted. A key feature of *Mtb* infection is the depletion of nicotinamide adenosine dinucleotide (NAD+), a central factor of metabolism, respiration, redox balance, and biosynthesis of molecular building blocks (82). This promotes *Mtb* pathogenesis and shaped drug design, as most TB drugs are NAD analogues or metabolized into NAD+ intermediates (83). NAD+ depletion is dependent on age and other conditions like obesity and type-2 diabetes, which are known risk factors for COVID-19 severity and mortality (84). Infection with SARS-CoV-2 also depletes cellular NAD+, which may be a primary determinant of the COVID-19 spectrum and a risk for mortality (85) suggesting that prior TB infection could enhance COVID-19 progression and vice versa.

Tuberculosis has been considered a risk factor for severe COVID-19 (86) with higher COVID-19 case fatality rates observed in areas like Peru with a high TB burden (87). Also, a case report of severe COVID-19 in an infant with disseminated TB has been reported (88). On a cellular level, the FCN1^+^/SPP1^+^ macrophage lineage is enriched in the lungs of severe COVID-19 patients. A study showed that some persons with active TB have increased levels of this macrophage lineage in circulation implying a risk of severe disease in TB/SARS-CoV-2 coinfection (89). *In vitro*, *Mtb* induces ACE2 and TMPRSS2 expression in macrophages leading to increased SARS-CoV-2 infection susceptibility, and consequently, *Mtb*-infected macrophages co-cultured with SARS-CoV-2 express a milieu of inflammatory response genes (89). Considered together, these factors suggest that there is a risk of severe disease in TB/SARS-CoV-2 co-infection. Contrarily, severe COVID-19 risk and mortality seem less in TB/COVID-19 co-infected patients in some studies (90, 91) indicating some protective effect of TB against severe COVID-19, but this needs to be specifically investigated. However, co-morbidities such as old age, respiratory diseases and diabetes contribute to increased severity and mortality in patients with COVID-19/TB co-infection (92, 93). A higher percentage of TB patients with COVID-19 have active pulmonary TB (91, 92, 94, 95) probably due to damage to the lungs making them more susceptible, nonetheless most patients present with mild symptoms with few reports of severe disease and death (66, 93). TB/COVID-19 co-infection leads to reduced SARS-CoV-2-specific immune response but increased TB-specific immunity without severe COVID-19 (96) meaning co-infection reduces COVID-19 immunity. The effect of COVID-19 on TB and vice versa is still unclear but it is important to tease out confounding factors that most likely contribute to the different outcomes in COVID/TB coinfections for better prognosis.

Immunity may be temporarily inhibited with the use of immunosuppressive drugs on SARS-CoV-2, resulting in latent *Mtb* activation and exacerbating the clinical outcomes (89). Primary TB protects the host by producing effective systemic immunity that prevents disseminated infection, while post-primary TB evades and distorts systemic immunity. As COVID-19 pathogenesis also depends on immune response, its severity may correlate with the phase of TB infection leading to an increase in TB burden as recorded in 2021 (33). This would necessitate a better understanding of these interactions to develop management strategies that will prevent a further upsurge of *Mtb* infection. The disease outcome and severity in patients with all three infections will far surpass that in an individual infected with either HIV or *Mtb*. Suffice to say that the above are predictions considering untreated patients. With treatment, COVID severity in TB and/or HIV-infected individuals may be significantly low.

### 2.3 Potential implications of COVID-19 on the control of TB and HIV/AIDS

The COVID-19 pandemic has caused an unprecedented interruption to health services, impeding the management of diseases like HIV/AIDS and TB. The urgent COVID-19 response posed competition in healthcare services, disrupted drugs and diagnostic kits supply chain, hindered access to healthcare and increased the burden on health practitioners leading to reduced clinical care because of the high influx of patients (97). This interruption has led to the reversal of global gains in combating HIV/AIDS and TB, especially in Africa (33). Also, the use of different drugs in patients with co-morbidities of SARS-CoV-2 with TB and/or HIV/AIDS could lead to drug-drug interactions, of which the immediate implications may not be seen. It could result in reduced or increased drug absorption, thereby modulating the action of drugs with adverse effects, reducing efficiency and increasing toxicity (98). Antivirals such as lopinavir, ritonavir, and remdesivir, used in COVID-19 therapy may interact with TB and/or HIV/AIDS treatments (99). Drug-drug interactions between protease inhibitors and rifampicin have been observed in HIV/AIDS and TB co-infection, reducing the efficiency of protease inhibitors (99), which may compromise COVID-19 treatment. New COVID-19 therapeutic approaches in HIV/AIDS or TB co-morbidities are needed to help tackle these diseases.

High-income or developed countries that mostly fund TB and HIV/AIDS control interventions have been severely affected by the COVID-19 pandemic and thus consigned their resources inward to alleviate the burden within their healthcare sectors. This jettisoned resources for research, and TB and HIV/AIDS control efforts in Africa. The Global Fund, to fight HIV/AIDS, TB, and other diseases, was also diverted to the COVID-19 response (100). Although the continuity of the fight against HIV/AIDS and TB has continued, the increase in SARS-CoV-2 transmission has reduced HIV/AIDS control in Africa, with high dependence on external partners for funding. Likewise, the enormous funds made available to combat COVID-19 in domains like research, therapeutics, diagnostics, personal protective equipment, construction, and hospital renovations have negatively impacted TB and HIV/AIDS funding in developing countries like Africa with a high HIV/TB co-infection burden. The question is, is the future somewhat bleak for the fight to eliminate TB and maintain a low HIV viral load in patients?

### 2.4 Management of TB and HIV amidst COVID-19 and stigmatization

Several antivirals have been incorporated into COVID-19 treatment and suppression (101, 102). Administering antivirals shortly after the onset of COVID-19 symptoms can reduce viral shedding, and prophylactic treatment of contacts could reduce infection risk (103). As high antivirals are needed for COVID-19 treatment with adequate stock of prophylaxis, two things are likely to happen: firstly, drug resistance and drug shortage due to increased usage. A few proposed COVID-19 drugs have antiviral resistance (104, 105) and overusing them could escalate this resistance, thus, rendering them useless. Drug resistance could result in increased morbidity and mortality, especially in viral and bacterial co-infected patients, including PLWH and TB. Secondly, drug overuse could aggravate both COVID-19 and AIDS conditions leading to complications. Continuous focus on COVID-19 at the expense of TB and HIV/AIDS might increase incidence and mortality rates in sub-Saharan Africa, already grappling with the burden of the earlier two epidemics. There is a significant risk of disruption of HIV/AIDS and TB treatments due to the diversion of efforts in combating COVID-19 (106). The continuous spread of COVID-19 in Africa stretches an already overwhelmed and inadequately resourced healthcare system (107) as TB incidence increases.

Thus, necessary measures need to be taken to ensure that combating COVID-19 is not at the expense of the gains made so far in HIV/AIDS and TB control. Ensuring a sufficient global supply of tests and treatment amidst the pandemic will be invaluable in sustaining TB and HIV/AIDS control interventions. Moreover, concurrent efforts in the management of these diseases are paramount to avoid exacerbating their morbidities and mortalities. Bulk distribution of HIV/AIDS and TB standard course medications could prevent treatment interruption due to efforts in combating the COVID-19 pandemic. Furthermore, proper and regular maintenance of testing equipment and facilities will help in sustaining efforts in the control of the three diseases. The provision of more diagnostic machines will equally be helpful to facilitate the workload.

Stigmatization is a discrediting social label that changes an individual’s self-image disqualifying them from full social acceptance (108). Stigma has been associated with HIV/AIDS and TB, worsening in HIV/TB co-infection (109). While ongoing efforts are made to tackle HIV/TB-associated stigma, there might be a shift to triple stigmatization with COVID-19. The stigma associated with COVID-19 is because of its novelty with many uncertainties and most infected individuals may not present for treatment, thereby continuing the spread of the pathogen (110). To reduce this triple stigma, modifications of policies and practices might be required while efforts are continuously made to reduce the spread of all three pathogens.

## 3 Conclusion

The COVID-19 response is unprecedented and has impacted TB and HIV/AIDS testing. Due to inadequate guidelines and patterns for SARS-CoV-2 co-infection, gleaning from past experiences with similar pathogens like SARS-CoV and MERS is of utmost benefit in tackling SARS-CoV-2 co-infections (111–116). Considering the inevitability of combined infections with HIV/AIDS and TB in Africa, comprehensive microbiologic surveys using syndromic multiplex panels and clinical evaluations are necessary. Also, research progress on HIV/AIDS and TB has been impeded, and clinical trials suspended in most countries as several researchers are redirected their efforts to COVID-19. However, increased collaboration with COVID-19 research has established new approaches to augment testing, and therapeutic and vaccine development, with potential long-term medical research advantages beyond the COVID-19 pandemic. Although COVID-19 vaccination is significantly high in many Western and Asian countries (117, 118), markedly changing the dynamics of the pandemic, repeated outbreaks and long-COVID-19 conditions are still being reported in several of these countries/regions. Thus, eradicating the disease seems to be hampered by inadequate knowledge and understanding of the SARS-CoV-2 biology, host immune response to the virus, COVID-19 treatment options, and vaccine efficacies and hesitancy, especially in Africa. Past investments in infectious disease training and research have produced remarkable gains in the COVID-19 response, indicating the need to retain them. Since several African countries have well-developed HIV and *Mtb* clinical trial infrastructure, it will be beneficial to incorporate SARS-CoV-2 in these collaborative efforts for a sustainable outcome to avoid losing ongoing gains, due to consequences of co-infections. Finally, adopting holistic medical care for COVID-19, HIV/AIDS, and TB co-infected patients is vital, with regular communications between TB/HIV programs and public health laboratories.

## Author contributions

AU conceptualized the study; AU, AD, KT, NN, FO, CA, and STE wrote the first draft of the manuscript; IAO and PQ edited and LP, GAA, and PKQ critically reviewed the manuscript. All authors contributed to the article and approved the submitted version.
